# Tailored nanophononic wavefield in a patterned bilayer system probed by ultrafast convergent beam electron diffraction

**DOI:** 10.1063/4.0000144

**Published:** 2022-06-03

**Authors:** N. Bach, A. Feist, M. Möller, C. Ropers, S. Schäfer

**Affiliations:** 1 Institute of Physics, University of Oldenburg, 26129 Oldenburg, Germany; 2 Max Planck Institute for Multidisciplinary Sciences, 37077 Göttingen, Germany

## Abstract

Optically excited nanostructures provide a versatile platform for the generation of confined nanophononic fields with potential (non-)linear interactions between different degrees of freedom. Control of resonance frequencies and the selective excitation of acoustic modes still remains challenging due to the interplay of nanoscale geometries and interfacial coupling mechanisms. Here, we demonstrate that a semiconductor membrane patterned with a platinum stripe acts as a tailored source for high-frequency strain waves generating a multi-modal distortion wave propagating through the membrane. To locally monitor the ultrafast structural dynamics at a specific distance from the deposited metal stripe, we employ ultrafast convergent beam electron diffraction in a laser-pump/electron-probe scheme. Experimentally observed acoustic deformations are reproduced by numerical simulations in a continuous medium model, revealing a spatiotemporal evolution of the lattice dynamics dominated by local rotations with minor strain and shear contributions.

## I. INTRODUCTION

In recent years, non-equilibrium excitations in solids were utilized to trigger phase transitions on ultrafast time scales and led to the discovery of novel transient phases and phase transition pathways.^1,2^ Efficient steering of solid-state systems into (meta-) stable states is typically achieved through direct coupling to the electronic subsystem.^3–15^

Light-driven optical^16–18^ and acoustic phonons^12,16^ as well as inhomogeneous nanoscale strain distributions^19,20^ have also been demonstrated to induce and modulate ultrafast phase transitions. However, it remains challenging to achieve detailed control of the spatiotemporal evolution of nanophononic wavefields. The underlying mechanisms of the optical excitation of acoustic phonons in nanoscale geometries^21^ can be revealed by experimental approaches that capture the ultrafast structural dynamics with a sufficient spatial and temporal resolution. Aside from the recent progress in ultrafast x-ray^22–26^ and extreme-ultraviolet scattering techniques,^27,28^ ultrafast electron probing approaches have been established for the investigation of structural dynamics. In particular, ultrafast transmission electron microscopy (UTEM) maps nanophononic fields^29–35^ harnessing diffraction contrast in bright- and dark-field imaging. A quantitative access to local structural distortions on fs- and ps-time scales is gained by ultrafast convergent beam electron diffraction (U-CBED), simultaneously probing the electron diffraction intensity at different incident angles. Previous U-CBED experiments explored phonon excitations in simple geometries^36–39^ but lacked the capability for spatially tailored sample excitations.

Here, we resolve the optically induced structural response of a thin silicon membrane in close proximity to a patterned platinum stripe by employing ultrafast convergent beam electron diffraction. A multi-modal crystal distortion wave is launched at the silicon/platinum interface and propagates through the silicon membrane. Time-resolved changes in the electron diffraction patterns are quantitatively reproduced in a continuous medium model. The evolution of the inhomogeneous distortion field is governed by a superposition of Lamb waves frequency-matched to local strain resonances in the bilayer region.

## II. ULTRAFAST CONVERGENT BEAM ELECTRON DIFFRACTION

**FIG. 1.**
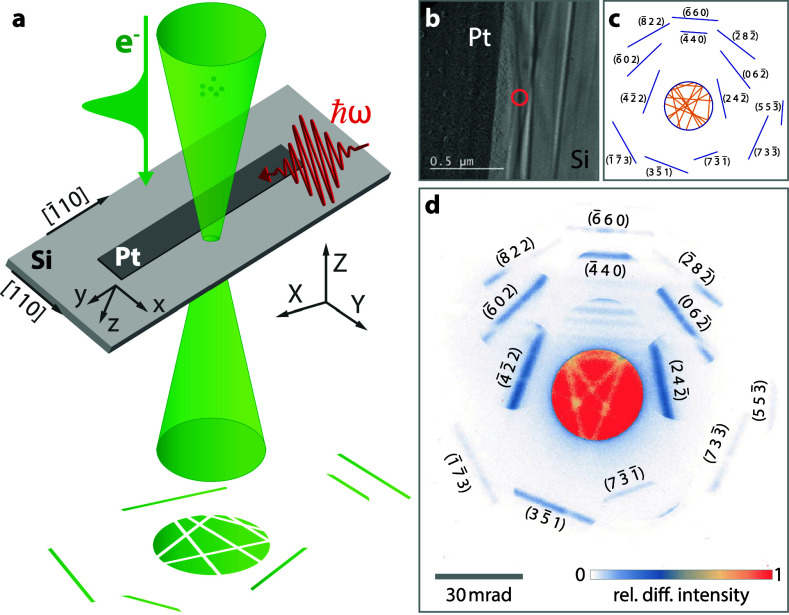
Ultrafast convergent beam electron diffraction on a Pt/Si heterostructure. (a) Experimental geometry of nanoscale diffractive electron probing on a single-crystalline silicon membrane (see arrows at membrane edges for crystalline orientation) in close vicinity to a polycrystalline platinum stripe. Inhomogeneous structural dynamics are induced by pulsed optical excitation of the platinum stripe. Laboratory- and sample-fixed coordinate systems are indicated by capital letters *X*, *Y*, and *Z* and lowercase letters *x*, *y*, and *z*, respectively. (b) Bright-field electron micrograph of the platinum stripe on the silicon membrane with the electron probing position (marked by red circle) in a distance of about 170 nm to the platinum stripe edge. (c) Calculated deficit (orange) and excess (blue) Bragg lines (labeled by Miller indices) for the employed sample orientation. (d) CBED pattern before optical excitation recorded at the probing position.

For a controlled generation of ultrafast structural distortion waves, we utilize optically excited nano-patterned semiconductor thin films. In such an approach, the geometry of deposited metal structures is expected to have a strong influence on the evolving spatiotemporal distortion field. In the present work, we prepared 2 *μ*m wide platinum stripes (10-nm thickness) on a (100)-oriented single-crystalline silicon membrane (35-nm thickness). Excitation of platinum by optical pulses (800-nm center wavelength) induces thermal stress within the stripe and initiates a strain wave propagating away from the platinum edge. The structural distortion is probed by focused ultrashort electron pulses (120-keV electron energy, sub-picosecond temporal duration^40^) in a distance of about 170 nm from the platinum stripe edge [Figs. 1(a) and 1(b)]. Specifically, we stroboscopically record diffraction patterns locally on the silicon membrane using a convergent electron beam (32-mrad full convergence angle, FWHM focal spot size of about 15 nm) for varying optical-pump/electron-probe delay times Δt. The incident cone of the incoming electron beam is represented by an intense central disk in the diffraction patterns. Owing to the broad angular distribution, multiple Bragg scattering conditions are fulfilled simultaneously.^41^ Exemplarily for a specific sample orientation, deficit intensity lines within and excess Bragg lines around the disk are displayed in Fig. 1(c) and 1(d). To obtain a larger set of distinct Bragg lines, the sample is tilted with respect to the electron beam direction. The position of each Bragg line is linked to a reciprocal lattice vector *G_hkl_* of the local (distorted) crystal structure of the silicon membrane. Thereby, *G_hkl_* is also related to the shape and orientation of the local real-space unit cell of silicon. Considering conservation of momentum and elastic scattering,^42^ as well as applying a paraxial approximation, the diffraction excess line positions in the detector plane are given by

$$r_{G}=\frac{-G_{\mathrm{hkl}}^{2}/2+k_{0}G_{Z}}{\sqrt{G_{X}^{2}+G_{Y}^{2}}}+\sqrt{G_{X}^{2}+G_{Y}^{2}}.$$ (1)

Here, *k*_0_ is the incident electron wave vector (oriented along the *Z*-direction) and *G_hkl_* is the reciprocal lattice vector in the laboratory-fixed coordinate system with Miller indices *h*, *k*, and *l* (see supplementary material S4 for details^51^). The inclination of the lines is characterized by an angle ϕ, given by $\text{tan}\;(\phi )=G_{X}/G_{Y}$. Based on the given scattering conditions,^43^ each Bragg line in the CBED pattern can be labeled with corresponding Miller indices [see Figs. 1(c) and 1(d)].

**FIG. 2.**
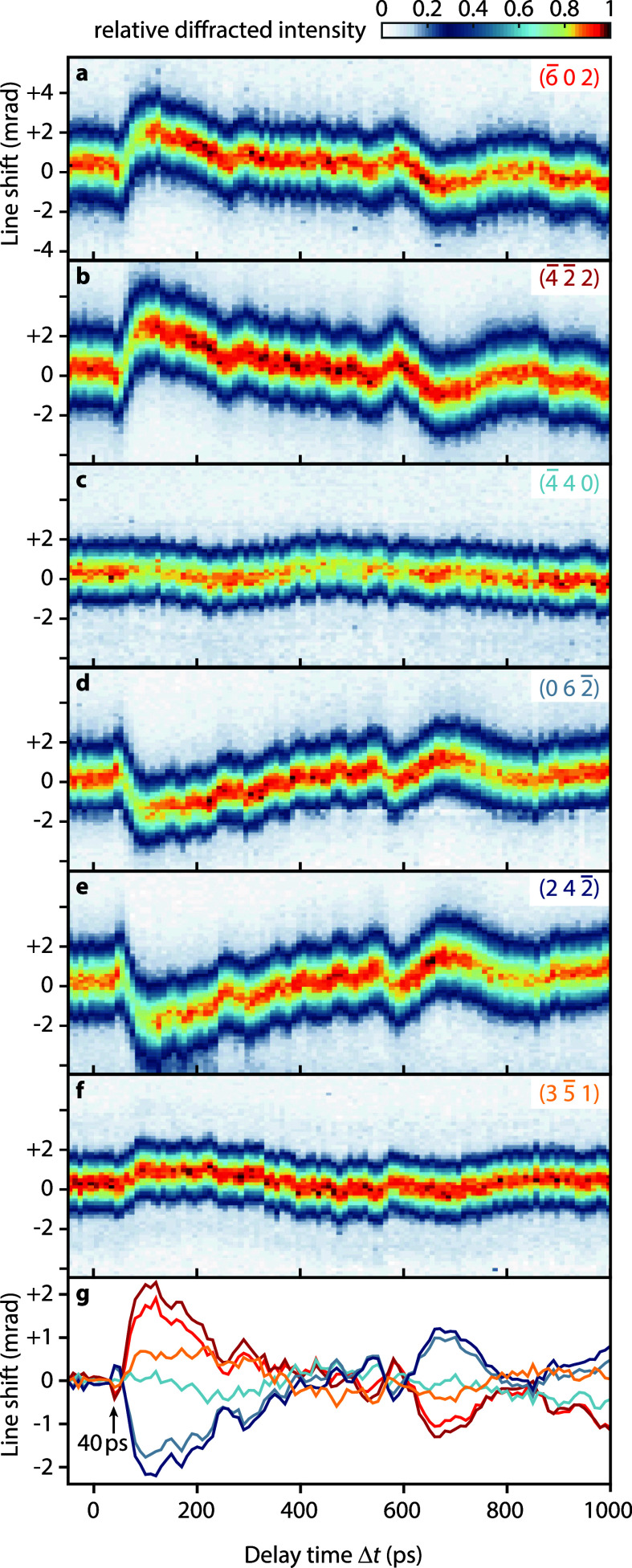
Transient Bragg line profiles. Delay-dependent profiles of selected Bragg lines recorded in the U-CBED experiment (a)–(f) and extracted peak shifts of Lorentz-profiles fitted to each Bragg line cross section (g). Each Bragg line map is normalized to its signal maximum.

Selected Bragg line profiles extracted from the ultrafast CBED patterns are shown in Fig. 2 as a function of the optical-pump/electron-probe delay time Δt. The transient changes of Bragg line profiles are obtained by integrating the diffracted intensity along the individual line directions. All profiles are background corrected and normalized to the intensity within the disk, as detailed in supplementary material S3.^51^ Depending on the Miller indices, the Bragg lines show varying delay-dependent angular shifts Δθ of up to 2 mrad. In all cases, shifts are only observed after an initial delay of about 40 ps due to the propagation time between the strain wave source and the electron probing position. The precise relative timing of electron pulse arrival at the sample and optical excitation was independently characterized by inelastic electron-light scattering.^44,45^ On a 500-ps time scale, the absolute line shifts exhibit an overall decrease with an additional complex temporal modulation containing multiple frequencies in the gigahertz range. These reproducibly observable small-amplitude oscillations become more evident from the delay-dependent central position, extracted from fitting Lorentz-profiles to each Bragg line cross section [see Fig. 2(g), for details of the analysis see supplementary material S3^51^]. After the zero-crossing of most line shifts at around 600 ps, further low-frequency oscillations are observed. Bragg lines corresponding to reciprocal lattice vectors pointing along the stripe, such as $\left(\bar{4}40\right)$ and $\left(3\bar{5}1\right)$ [see Figs. 2(c) and 2(f)], show a largely reduced angular shift. This selective behavior is expected due to the translational-symmetric strain field along the long stripe ([ $\bar{1}10$]-direction). A further peculiar feature in the line profiles is the decrease in the scattering intensity maximum in most selected lines around 80 ps. This intensity change indicates an inhomogeneous strain profile within the depth of the silicon membrane as previously observed in similar studies for an optically excited graphite flake.^37^

## III. NUMERICAL SIMULATION OF NANOPHONONIC DISTORTION FIELD

To identify the origin and analyze the spatiotemporal evolution of the strain dynamics in the Pt/Si heterostructure, we numerically solve the anisotropic elastodynamic wave equation using a finite-element approach. Due to the symmetry of the problem, we implement a two-dimensional *x*–*z* cross section with an infinitely extended platinum stripe in the *y*-direction [for coordinate system, see Fig. 1(a)]. Considering the stripe length (100 *μ*m), probing position (approximate distance from stripe corners 50 *μ*m), and silicon strain wave velocities^46^ ( $v_{l}=8432\;{\text{ms}}^{-1}$), lattice distortions from the stripe corners do not affect the structural dynamics in the sub-6-ns range. The optical excitation of the platinum stripe is implemented as a depth-dependent heat source adapted to the absorbed local optical power in the experiment. The thermal coupling between the platinum layer and the silicon membrane is determined by the thermal boundary resistance (see supplementary material S7 for details^51^).

**FIG. 3.**
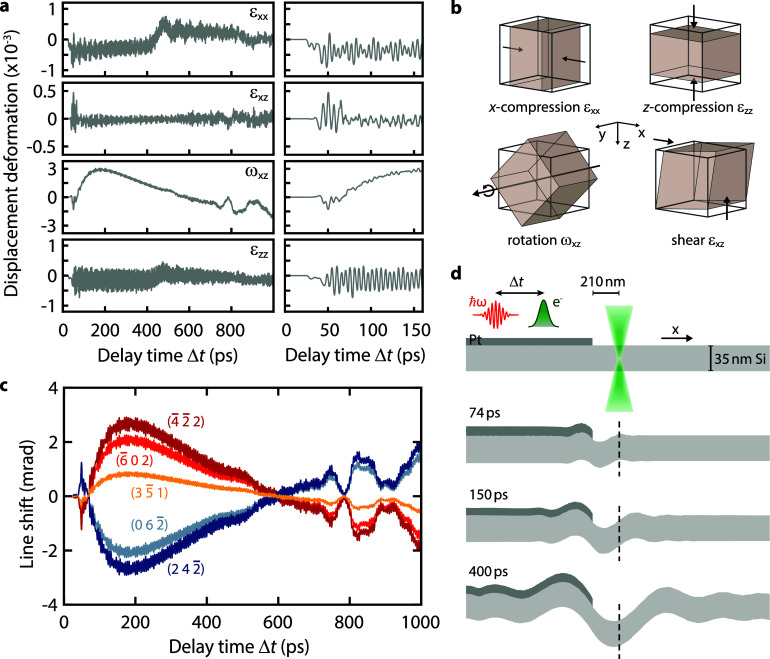
Simulated acoustic wave dynamics close to the Pt-stripe/Si-membrane heterostructure. (a) Temporal dynamics of selected strain and rotation tensor components extracted at a distance of 210 nm from the Pt stripe edge. Each component is averaged along the *z*-direction. Close-ups for early delay times (displayed in the right column) reveal multi-frequency oscillations. (b) Schematic representation of lattice distortions associated with the selected deformation gradient tensor components shown in (a). (c) Predicted Bragg line shifts based on the simulated wave dynamics taking into account the four symmetry-allowed tensor components. (d) Real-space distortion of silicon and platinum surfaces before and after optical excitation at different delay times. For better visibility, the surface displacements are scaled by a factor of 250. A large wavelength overall membrane bending is superimposed by additional high-frequency oscillations with small amplitudes.

Generally, the local lattice distortion can be described by a time-dependent deformation gradient tensor^43^ F(Δt)=ϵ(Δt)+ω(Δt)+1, where *ϵ* and *ω* are the symmetric strain and antisymmetric rotation tensors, respectively. Due to the effective symmetry of the problem, *F* can be represented by a 2 × 2 matrix with components *F_ij_* [ i,j∈(x,z)]. The diagonal entries *F_xx_* and *F_zz_* signify compression or dilatation along the respective directions. The off-diagonal components *F_xz_* and *F_zx_* describe a shearing [ $\varepsilon_{xz}=(F_{xz}+F_{zx})/2$] and a rotation [ $\omega_{xz}=(F_{xz}-F_{zx})/2$] of the unit cell. A schematic representation of the associated lattice distortions is given in Fig. 3(b). From the numerically simulated temporal evolution of the tensor components for our sample system [see Fig. 3(a)], it is apparent that the main lattice distortion induced by the optically illuminated platinum stripe consists of a local rotation of the unit cell and only smaller contributions from the strain tensor. High-frequency oscillations are observed in all components, as visible in the right panels of Fig. 3(a).

For visualizing the optically induced membrane dynamics, we extracted the time-dependent displacement vector component *u_z_* at each point of the material layer surfaces and the interface of the Pt/Si heterostructure and constructed the displaced surfaces at four delay times in Fig. 3(d). For enhanced visibility, the displacement is scaled by a factor of 250. Two mechanisms are responsible for the observed deformation of the membrane, both linked to the optically induced heating of the platinum stripe. First, thermal expansion of the stripe in the transverse (*x*-) direction results in a bimetal-like bending of the underlying silicon membrane [Fig. 3(d)]. The detailed shape of the resulting membrane bending wave is expected to be governed by the time scale of the platinum expansion and the mechanical coupling between platinum and silicon. Second, additional fast small-amplitude displacement oscillations [Fig. 3(a)] are induced by local resonances in the bilayer region resulting in a superposition of symmetric and antisymmetric Lamb waves with a range of frequencies *ω* as discussed below. At the electron probe position next to the platinum stripe, the main displacement results from the overall bending of the membrane encoded in the rotation tensor *ω_xz_*.

From the numerically obtained tensor components *F_ij_*, the temporal shift of the expected Bragg line positions *r_G_* can be calculated (see supplementary material S4^51^). Choosing the experimentally selected Bragg lines and time-dependent tensor components at a distance of 210 nm to the platinum stripe (similar to the experimental probe position of about 170 nm) yields the results shown in Fig. 3(c). The numerical line shifts closely resemble the experimental findings with few-percent deviations particularly in the first 160 ps (see Fig. S2 in the supplementary material for details^51^), providing a close link between the simulation and the experiment. Only the dynamics of the ( $\bar{4}40$) and ( $\bar{6}60$) lines are not recovered, indicating a dependence on omitted tensor components containing deformations along the *y*-axis. Deviations from the behavior expected for reasons of symmetry may result either from an overall deformation occurring during the deposition of the platinum stripe or from local membrane distortions induced by the mechanical contact with the polycrystalline stripe.

Whereas the simulated tensor components can be used to predict Bragg line shifts [cf. Eq. (1) and supplementary material S4^51^], the full reconstruction of the tensor components from the experimental data is generally not achievable. For a detailed analysis of the tensor component subspace which is experimentally accessible, we consider a linearized relation between the Bragg line positions and the tensor components. The corresponding derivatives $\frac{\partial r_{G}}{\partial F_{ij}}$ are collected in a sensitivity matrix *A* (see supplementary material S5^51^). A singular value decomposition of *A* reveals three linear combinations of tensor components which the U-CBED experiment is most sensitive to. In the lab coordinate system, the eigenvectors correspond to displacement gradients along the direction of the incoming electron probe pulses. For the chosen sample orientation and the selected Bragg-scattered lines, we obtain a high sensitivity to two shear-rotational motions perpendicular to the *z*-axis and to vibrations along the *z*-axis. This result is consistent with the general sensitivity of line shifts in CBED experiments to changes of reciprocal lattice vectors along the electron beam direction (see supplementary material S5^51^).

## IV. LAMB WAVE DECOMPOSITION

**FIG. 4.**
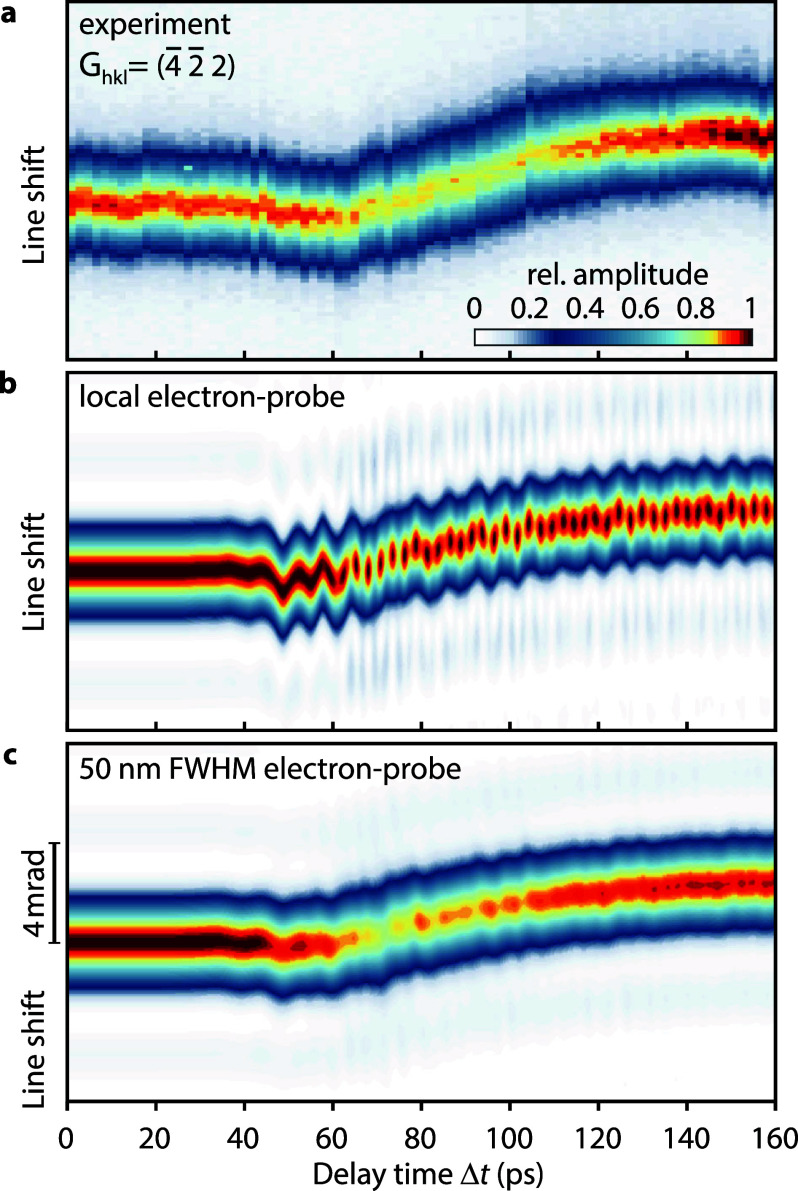
Temporal evolution of Bragg line profile. (a) Experimental time-dependent cross sections of the ( $\bar{4}\bar{2}2$) line show a decrease in the scattered intensity maximum between 70 and 100 ps. Calculated line profiles using numerically simulated displacement fields for (b) a local electron probe with negligible spatial extension and (c) considering spatial averaging due to an electron beam focused to 50 nm FWHM.

Apart from the transient shift of selected Bragg-scattered lines, most lines exhibit a pronounced drop in the intensity maximum in the line profile around 80 ps. For a detailed analysis of the profiles, we recorded an additional delay scan with 2-ps time steps. Cross sections through the $\left(\bar{4}\bar{2}2\right)$ line are shown in Fig. 4(a). The line profiles in CBED patterns are related to the inhomogeneity of the displacement field, both within the electron spot diameter and along the depth of the thin film.^22,43^ The strained crystal imprints a phase modulation on the electron exit wave resulting in a diffracted intensity $I_{\mathrm{hkl}}\propto |f\left[\text{exp}\;(2\pi i\;G_{\mathrm{hkl}}\cdot u(r))\right]{|}^{2}$. Due to the different length scales involved, we chose two approaches to include the effects in our calculations. While we treat the displacement inhomogeneity within the depth of the membrane explicitly, we assume a spatial average for the transverse variations of the electron probe.

In the limit of a perfectly localized electron probe, the simulated line profiles, as shown in Fig. 4(b), largely reproduce the experimentally observed linewidth and the temporal variations in the maximum line amplitude and line position. Interestingly, and different from the experiment, the maximum intensity in the line profiles oscillates with a period of 2.4 ps, visible particularly for delay times above 60 ps. Within the oscillation, the associated intensity is relocated to the diffraction sidebands at angular distances similar to the first maximum of the thin-film shape function (see supplementary material S6 for details^51^). Considering, additionally, the incoherent averaging due to a 50 nm FWHM lateral size of the electron probe beam, the oscillatory features are averaged out, as shown in Fig. 4(c), and a closer match with the experimental results is obtained. We note that the experimental line profiles do not show scattering sidebands that clearly exceed the background level [except for the partially visible $\left(\bar{2}20\right)$-disk in Fig. 1(d)]. This effect arises from an additional line blurring by diffuse and inelastic scattering processes.

**FIG. 5.**
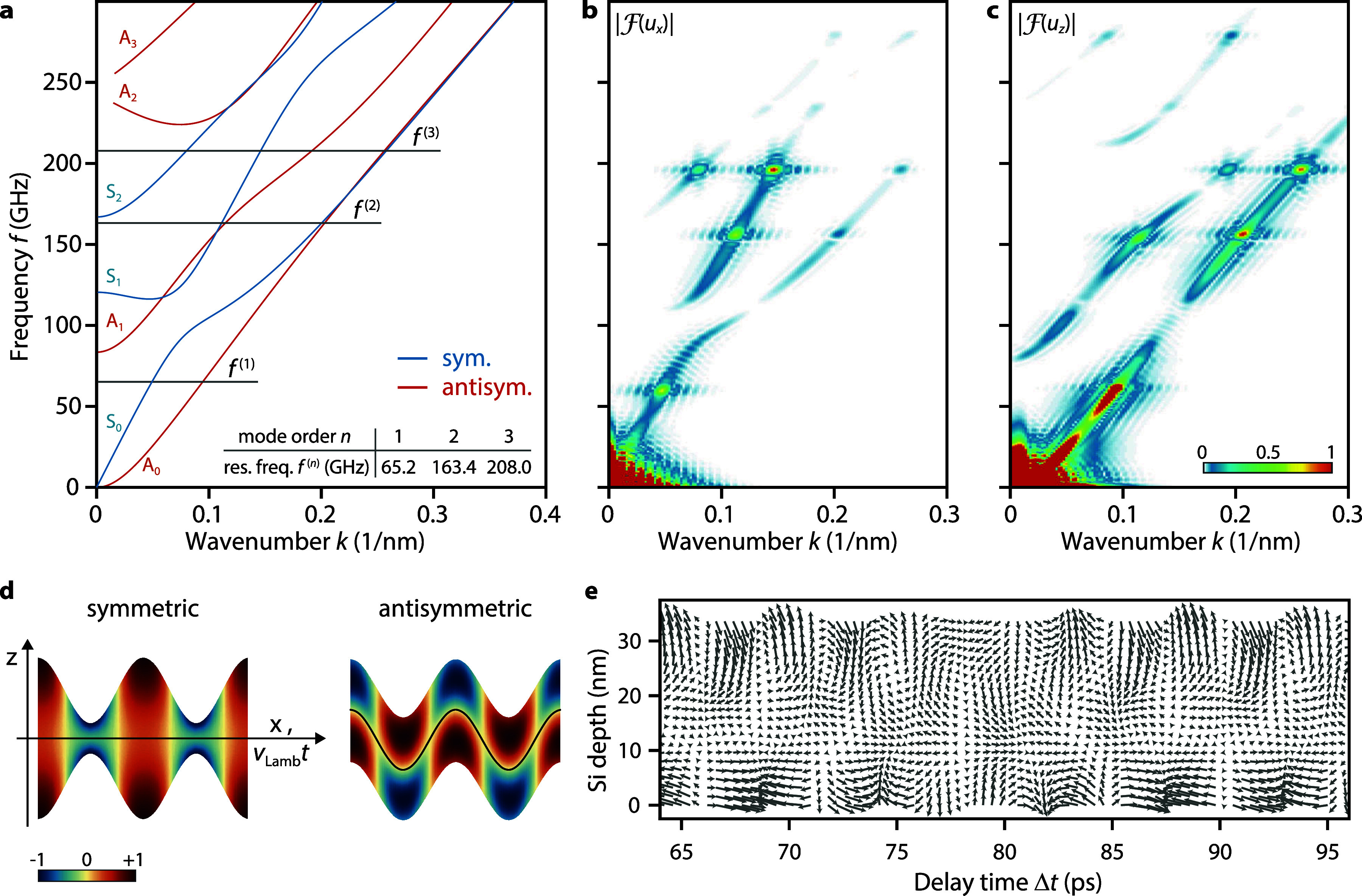
Mode decomposition of the acoustic wave field. (a) Lamb dispersion curves for a 35-nm thin silicon membrane with k∥[110], decoupled into symmetric S_*n*_ and antisymmetric A_*n*_ modes. As a reference, the three lowest resonance frequencies $f^{\left(n\right)}$ in a homogeneous Pt/Si bilayer system are given. Spatiotemporal Fourier decomposition of the mean displacements *u_x_* (b) and *u_z_* (c), obtained from finite-element simulations. Prominent amplitude maxima appear at the resonance frequencies of the Pt/Si bilayer system. (d) Schematics of displacement field for the symmetric S_1_ and antisymmetric A_1_ Lamb modes at $f^{\left(2\right)}=163.4\;\text{GHz}$. ( $u_{z,\text{Lamb}}$ is indicated as a scaled surface displacement. Color-coding corresponds to $u_{x,\text{Lamb}}$.) (e) Vector field representation of the superposition of selected Lamb modes qualitatively reproducing the numerically simulated displacement fields (cf. Fig. 6).

Finally, we discuss the microscopic origin of the overall membrane bending and the high-frequency oscillations obtained in the finite-element simulation. Generally, the acoustic spectrum in a planar wave guide consists of an infinite number of propagating waves, each characterized by the in-plane wave vector *k*, a vectorial mode profile and dispersion relations ω(k). For the single-crystalline silicon membrane studied here with lattice distortions restricted to the *x*–*z*-plane (spanned by the [110]- and [001]-crystalline directions, i.e., sagittal polarization), acoustic modes decouple into pure Lamb modes.^47–49^ They are designated S_*n*_ and A_*n*_ based on the associated displacements which are symmetric or antisymmetric relative to the mid-plane of the membrane. The corresponding dispersion curves for the current geometry are displayed in Fig. 5(a) (see supplementary material S9 for details on dispersion curve tracing and calculation of displacements^51^). The mode index $n\in {\mathbb{N}}_{0}$ signifies the number of nodes of the displacements along the out-of-plane *z*-direction. Figure 5(d) depicts an example for the displacement field for both an A_1_ and S_1_ wave at a frequency of 163.4 GHz and corresponding wavenumbers as extracted from the dispersion relation.

**FIG. 6.**
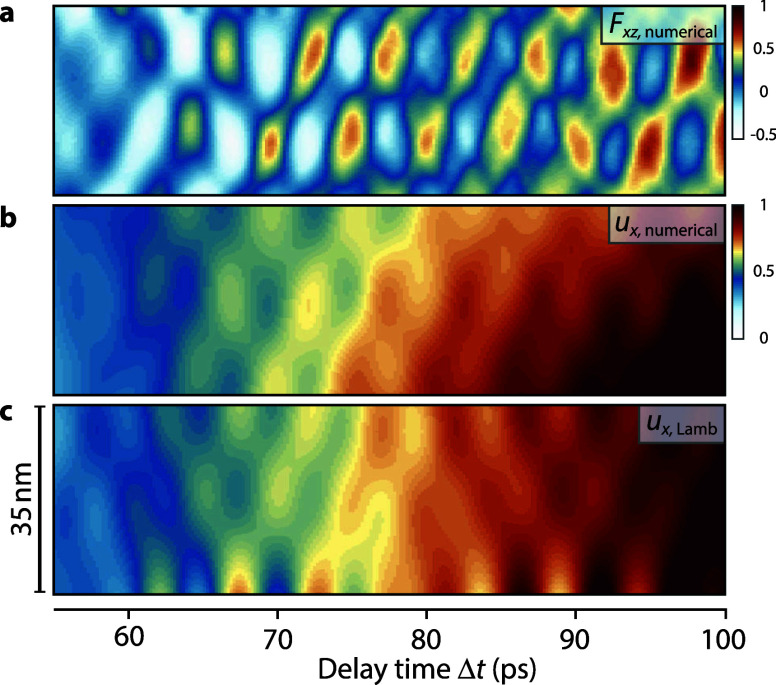
Spatiotemporal dynamics of the acoustic wave field. (a) and (b) Calculated evolution of the shear-rotation tensor component *F_xz_* and corresponding displacement field *u_x_* at a distance of 210 nm from the platinum stripe edge. (c) Displacement field obtained from a Lamb mode superposition in *x*-direction with additional contributions from the numerical through-thickness averaged displacement, qualitatively reproducing the results in (b).

In Figs. 5(b) and 5(c), we show the decomposition of the numerically simulated strain wave profile into Lamb wave amplitudes revealing a strong excitation of the *A*_0_ (flexural) Lamb mode in the low-frequency regime ( <40 GHz). Additional local maxima in the amplitude of the zero-order branches, as well as on S_*n*_ and A_*n*_ branches with mode order *n *> 0, appear at specific higher frequencies. These frequencies are linked to quasi-standing acoustic resonances in the Pt/Si bilayer region which acts as the source of the Lamb wave field. Within an analytical acoustic mode description of resonant longitudinal strain modes,^50^ the lowest resonance frequencies in the Pt/Si bilayer system are located at 65.2, 163.4, and 208.0 GHz [horizontal lines in Fig. 5(a)]. These distinct resonances are in good agreement with the excitation spectrum observed in Figs. 5(b) and 5(c). Similarly, for delay times above 60 ps, the bilayer resonance frequency at 208 GHz is also visible in the tensor component *F_xz_*, as shown in Fig. 6(a), as well as in the simulated diffraction line profiles in Fig. 4(b). In accordance with these results, the complete displacement wave field can be well reproduced by just considering a superposition of Lamb waves at the bilayer resonance frequencies and an additional low-frequency component taken from the average of the simulated displacement field at each delay time [for the *u_x_* component see Figs. 6(b) and 6(c), *u_z_*: Fig. S2]. For an alternative representation of the obtained displacement field, see Fig. 5(e).

The characteristics of the induced acoustic wave field can be tuned by the metal/semiconductor bilayer structure. In particular, changing the impedance matching or the individual thicknesses allows for tuning of frequency components in the wave field. Furthermore, while in the current geometry Lamb waves with a broad range of wavevectors along the *x*-direction are excited, tailored coupling schemes of the acoustic wave source to the membrane may lead to the generation of Lamb wave superpositions with a narrowband wavevector distribution.

## V. CONCLUSION

Our results demonstrate that patterned semiconductor membranes are a versatile platform for optically inducing nanoscale acoustic waveforms. The propagating wave, as experimentally probed by ultrafast convergent beam electron diffraction, is composed of Lamb waves triggered by local resonances in the structured area of the membrane. The presence of small-amplitude symmetry-forbidden distortions already points to the importance of local membrane defects and inhomogeneous nanoscale pre-straining.

Since the explicit patterned geometry and its coupling to the homogeneous membrane have a strong impact on the generated acoustic wavefield, the chosen model heterostructure offers various opportunities for further tailoring and locally enhancing the structural response via acoustic wave interferences. In particular, acoustic foci may allow for an ultrafast control of local strain-driven phase transitions.

## Data Availability

The data that support the findings of this study are available from the corresponding author upon reasonable request.
